# Additional Observations on Croup

**Published:** 1842-02-19

**Authors:** Caspar Morris


### MEDICAL EXAMINER.

NEW SERIES.

No. 8.] PHILADELPHIA, FEB. 19, 1842. [Vol. I.

### ORIGINAL COMMUNICATIONS.

### *Additional observations on Croup.*

By Caspar Morris, M. D.

[The following additional observations were not received until the paper of Dr. Morris, published in our last number, was in type. They may be considered as an appendix to that paper.]

I some time since examined the body of a child who died of this disease, under the care of one of my friends, and found the upper portion of the trachea free from lymph, but much inflamed, while the most minute ramifications of the bronchii in both lungs were coated with exudation. This fact alone is sufficient to induce a doubt of the value of the operation of laryngotomy. Various devices have been suggested, especially by the French authors, who speak of clearing the respiratory tubes pretty much as they would of sweeping a chimney, for removing the effused lymph; but the same objection lies to this operation as was suggested before. The diseased condition of the membrane still remains. A case recently fell under my observation which terminated fatally. A child of two years, recovered from an attack of scarlet fever only two months, was taken with the common symptoms of croup. I did not see it during the first twenty-four hours. I was sent for on the second evening, and found it with a hoarse but not very dry cough; voice whispering; no great dyspnœa. It was late in the evening when I saw it; and as there were but little fever and no urgent symptoms, I ordered an emetic, leaving directions to be called in the night in case it did not act or afford relief. In the morning I found, by the report of the mother, that the emetic had operated, affording great relief, but that the symptoms had returned at an early hour in the morning. At my visit the child was hoarse with a dry resonant cough; hot skin and quick feeble pulse. Upon examining the tonsils they were found completely coated with lymph; blood was taken from the arm, and the treatment I have suggested pursued. Large quantities of lymph were thrown off under the influence of the tartrate of antimony and calomel, and the child was much relieved. A relapse, however, took place on the third day, and it was again bled and the treatment resumed. During the first attack a blister had been formed by the application of the caustic solution of ammonia. This now took on the sloughing process, and in despite of my efforts the child sunk under the irritation, retaining the peculiar voice and cough to the last. Upon examination, the larynx and trachea exhibited decided traces of inflammation, but the whole membrane had been evacuated. Upon what did the cough, the peculiar voice, and the strangulation depend in this case? There was no thickening; no deposits remaining; and the condition of the ventricles was perfectly healthy. Never having met with epidemic croup, I have purposely avoided any reference to it in a lecture intended merely to present a few views growing out of my own observation.

